# Electronic Health Record and Semantic Issues Using Fast Healthcare Interoperability Resources: Systematic Mapping Review

**DOI:** 10.2196/45209

**Published:** 2024-01-30

**Authors:** Fouzia Amar, Alain April, Alain Abran

**Affiliations:** 1 École de technologie supérieure - ETS Montreal, QC Canada

**Keywords:** electronic health record, EHR, Health Level Seven International Fast Healthcare Interoperability Resources, HL7 FHIR, interoperability, web ontology language, OWL, ontology, semantic, terminology, resource description framework, RDF, machine learning, ML, natural language processing, NLP

## Abstract

**Background:**

The increasing use of electronic health records and the Internet of Things has led to interoperability issues at different levels (structural and semantic). Standards are important not only for successfully exchanging data but also for appropriately interpreting them (semantic interoperability). Thus, to facilitate the semantic interoperability of data exchanged in health care, considerable resources have been deployed to improve the quality of shared clinical data by structuring and mapping them to the Fast Healthcare Interoperability Resources (FHIR) standard.

**Objective:**

The aims of this study are 2-fold: to inventory the studies on FHIR semantic interoperability resources and terminologies and to identify and classify the approaches and contributions proposed in these studies.

**Methods:**

A systematic mapping review (SMR) was conducted using 10 electronic databases as sources of information for inventory and review studies published during 2012 to 2022 on the development and improvement of semantic interoperability using the FHIR standard.

**Results:**

A total of 70 FHIR studies were selected and analyzed to identify FHIR resource types and terminologies from a semantic perspective. The proposed semantic approaches were classified into 6 categories, namely mapping (31/126, 24.6%), terminology services (18/126, 14.3%), resource description framework or web ontology language–based proposals (24/126, 19%), annotation proposals (18/126, 14.3%), machine learning (ML) and natural language processing (NLP) proposals (20/126, 15.9%), and ontology-based proposals (15/126, 11.9%). From 2012 to 2022, there has been continued research in 6 categories of approaches as well as in new and emerging annotations and ML and NLP proposals. This SMR also classifies the contributions of the selected studies into 5 categories: framework or architecture proposals, model proposals, technique proposals, comparison services, and tool proposals. The most frequent type of contribution is the proposal of a framework or architecture to enable semantic interoperability.

**Conclusions:**

This SMR provides a classification of the different solutions proposed to address semantic interoperability using FHIR at different levels: collecting, extracting and annotating data, modeling electronic health record data from legacy systems, and applying transformation and mapping to FHIR models and terminologies. The use of ML and NLP for unstructured data is promising and has been applied to specific use case scenarios. In addition, terminology services are needed to accelerate their use and adoption; furthermore, techniques and tools to automate annotation and ontology comparison should help reduce human interaction.

## Introduction

### Background

The development and deployment of electronic health records (EHRs) worldwide has led to several interoperability challenges, including extracting valuable information from free-text clinical notes, integrating unstructured EHR patient data, and reusing them without ambiguity for clinical research purposes [1,2].

**Figure 1:**
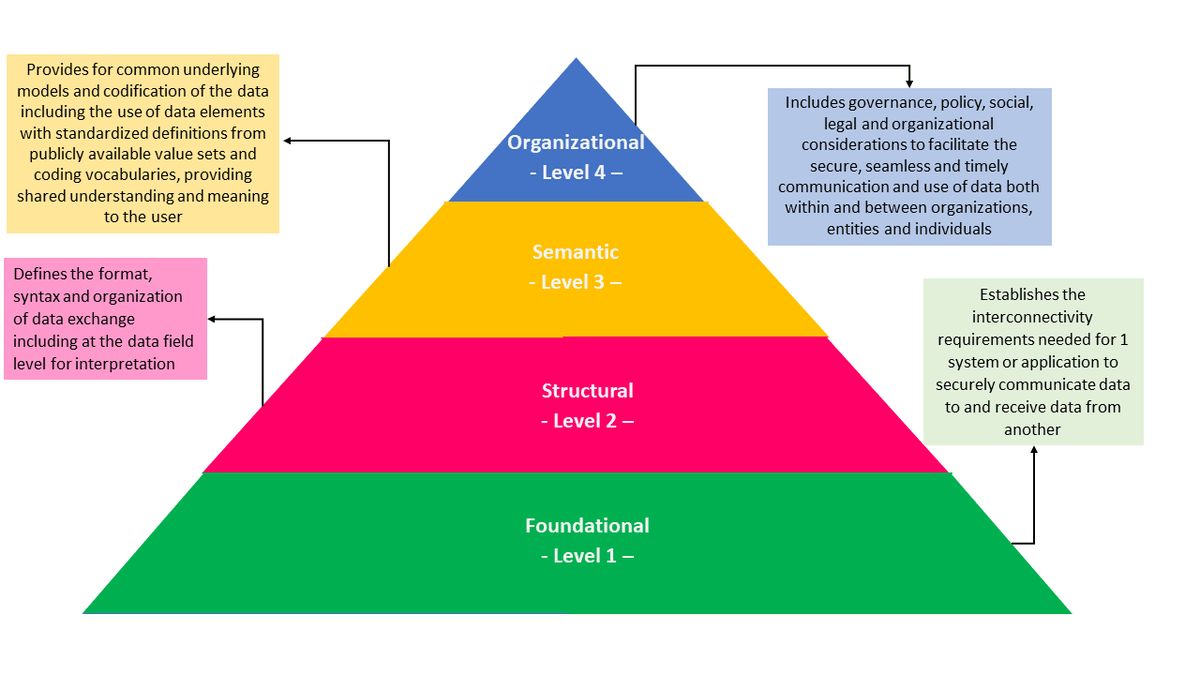
Interoperability levels (Healthcare Information and Management Systems Society).

Interoperability is defined by the Institute of Electrical and Electronics Engineers (IEEE) as the “ability of two or more components to exchange information and to use the information that has been exchanged” [3]. A total of 4 interoperability levels were proposed by the Healthcare Information and Management Systems Society organization: (1) foundational, (2) structural, (3) semantic, and (4) organizational (Figure 1) [4].

Important research efforts are underway to develop health care standards, including Fast Healthcare Interoperability Resources (FHIR) standards for the electronic exchange of health care information. This standard was developed by the Health Level Seven International (HL7) organization aiming at the evolution of messaging standards to achieve semantic interoperability [5]. FHIR combines the best features of HL7 version 2, version 3, and clinical document architecture, while leveraging the latest web standards.

FHIR interoperability solutions are designed from a set of modular components called “resources” that have been designed as small reusable components defining a set of properties for the capture of the structure of the domain data acquisition for a particular use. The version 4.3.0 release of FHIR R4B proposes 143 different “resources” classified into five categories to handle health data: (1) foundation resources, (2) base resources, (3) clinical resources, (4) financial resources, and (5) specialized resources (Multimedia Appendix 1).

Many elements in FHIR use coded values and allow the use of external terminologies, such as (1) Systematized Nomenclature of Medicine–clinical terms (SNOMED CT)—comprehensive multilingual clinical terminology used in EHR. Its components include concepts (codes), descriptions (terms), and relationships—and (2) Logical Observation Identifiers Names and Codes (LOINC), a set of universal names and ID codes used to identify laboratory and clinical test results.

Common terminology aims to address the problem of ambiguity in exchanged data [6]. However, most legacy systems do not take advantage of a terminology-based approach to data exchange and still use incompatible terminologies or unstructured data in their exchanges with EHR. Thus, to ensure semantic interoperability with these legacy applications, it is mandatory to perform manual data transformations, mapping, or other alternate solutions to ensure that the exchanged data are interpreted correctly by the information systems involved.

The FHIR standard is of great interest to researchers and EHR ecosystem developers [7], and several technology giants, such as Amazon, Google, IBM, Microsoft, Oracle, and Salesforce, offer ready-to-use cloud services to promote the adoption of FHIR [8].

In this study, we report on a systematic mapping Review (SMR) and propose a classification of the FHIR literature based on FHIR resources, terminologies, research approaches, and innovative contributions. The goal of this SMR was to provide state of the art, trends, and analysis of research related to semantic interoperability using FHIR. This activity specifically aimed to identify the FHIR and terminology used, as well as the different proposed approaches, contribution types, and trends.

### Related Work

Of the number of literature reviews on the challenges of health information systems interoperability, only 4 specifically address FHIR standard issues [2,9-11]. This section summarizes the findings of this study.

The study by Ayaz et al [10] reviewed 80 studies published between 2012 and 2019 that identified the opportunities and challenges associated with using FHIR. Another FHIR systematic review by Lehne et al [9] identified the main issues to be considered when exchanging data based on only 2 data sources and presented an overview of 131 selected studies up to February 4, 2019. A third systematic literature review by Setyawan et al [2] on data interoperability issues and possible solutions selected 39 studies published between 2018 and 2021 from 3 data sources (ie, IEEE Xplore, ScienceDirect, and Scopus). Finally, a systematic review of semantic interoperability in the study by de Mello et al [11] selected 28 studies from 2012 to 2020 from 8 different sources.

An overview of these 4 studies is presented in Table 1 in terms of their study goal or objective, period covered, number of studies selected and analyzed, source databases, and their respective mapping and research questions (RQs).

**Table 1:** Overview of the 4 literature reviews on semantic issues when using Fast Healthcare Interoperability Resources (FHIR).

ID	Study, year	Goal or objective	Period covered	Studies, n	Source databases	Mapping and research questions
R1	Ayaz et al [10], 2021	A systematic review of the literature related to FHIR, including the challenges, implementation, opportunities, and future FHIR applications	January 2012-December 2019	80	ACM IEEE Springer Google Scholar PubMed ScienceDirect	Q1: Types or models of FHIR implementation? Q2: Common resources used in FHIR implementation? Q3: Applications that benefit from the use of FHIR? Q4: Approaches applied to map or migrate data from previous standards to FHIR? Q5: Goals of FHIR? Q6: Challenges and open questions related to the FHIR domain?
R2	Lehne et al [9], 2019	Screen all FHIR publications and identify the main topics	February 4, 2019	131	Web of science PubMed	N/A^a^
R3	Setyawan et al [2], 2021	Identify integration problems of HL7^b^ FHIR implementation and provide technical solutions and nontechnical solutions to these problems	2018-2021	39	ScienceDirect IEEE SCOPUS	Q1: State of the art of data integration and interoperability in HL7 FHIR implementation? Q2: Problems the researcher has regarding Data Integration and Interoperability in HL7 FHIR Implementation? Q3: Potential solutions and future research regarding Data Integration and Interoperability in HL7 FHIR Implementation?
R4	de Mello et al [11], 2022	Present a comprehensive systematic literature review of semantic interoperability in electronic health record	2010-2020	28	ACM IEEE Xplore Google scholar MEDLINE PubMed ScienceDirect SpringerLink Web of Science	Q1: State of the art in health standards applied in health records? Q2: Challenge and open questions to semantic interoperability in health records. Q3: Health standards adopted in the studies? Q4: Terminologies or health repositories used? Q5: Approaches used? Q6: Main security concerns used? Q7: Evaluation approaches used?

^a^N/A: not applicable.

^b^HL7: Health Level Seven International.

To the best of our knowledge, no systematic mapping review has been published that specifically addresses the semantic issues raised when using FHIR.

Some of the lessons learned in these studies and their limitations are presented in Table 2. The key findings are summarized as follows:

- Most used FHIR resources from general FHIR implementation up to 2019: Observation, Patient, Practionner, Condition, Medication, AllergyIntolerance, Medication, Device [10].
- The study by Ayaz at al [10] provides a distribution of the different techniques used for the mapping approach (eg, mapping HL7 v2 to FHIR and mapping FHIR to others).
- The formula string using FHIR as the keyword is general and allows maximum results [9]. This study showed that ontology or terminology approaches were subject to a higher number of studies up to 2018.
- One study [2] used a limited number of databases (only 3), and the formula string may limit the obtained results.
- We learned from the study by de Mello et al [11] that the main approaches used are based on ontology and terminology, particularly SNOMED CT, International Classification of Diseases (ICD)-9 or ICD-10.

**Table 2:** Lessons learned and limitations of related work.

ID	Lessons learned	Limitations
R1	Interesting mapping framework OR data model into the category of the most selected studies Common resources used	More databases are needed Formula for search may limit the result to semantic FHIR^a^ studies Are the identified resources still of interest for semantic interoperability? Mapping to terminologies is not covered
R2	Formula used help to select the maximum number of studies Topics of interest: ontology, terminology, data models ontology, terminology, data, and information model, mapping The ontology, terminology, and data models is the subject of a high number of the selected studies	Limited list of databases The number of studies continues to increase as of 2018
R3	N/A^b^	Limited database sources The search formula may limit the result as it must have the following: (challenge* OR problem* OR issue* OR trend*)
R4	Period: 2010-2020 Different approaches based on ontology and the use of terminology, especially SNOMED CT^c^, ICD^d^-9 or ICD-10 The use of OWL^e^ and RDF^f^ to solve semantic issues	HL7^g^ FHIR is not in the list of identified standards based on the selected studies The search based on semantic interoperability may limit the result as it used: “semantic interoperability” AND (“health record” OR “medical record” OR “patient record” OR “hospital record”) AND standard A small number of articles selected

^a^FHIR: Fast Healthcare Interoperability Resources.

^b^N/A: not applicable.

^c^SNOMED CT: Systematized Nomenclature of Medicine–clinical terms.

^d^ICD: International Classification of Diseases.

^e^OWL: web ontology language.

^f^RDF: resource description framework.

^g^HL7: Health Level Seven International.

These previous studies do not provide an overview of post-2019 developments related to semantic interoperability approaches and trends using FHIR. An extensive and more recent study is needed using many databases and a search string that includes the most possible semantic keywords.

In contrast to related works, this SMR focuses on the semantic level of interoperability using FHIR and reports on research directions over the last decade.

## Methods

### Overview

SMRs and systematic literature reviews typically use the same methodology, but their goals differ. The systematic literature review is driven by specific questions and the review aims to respond to these specific questions. In contrast, an SMR targets more high-level questions aimed at a specific topic, with the goal of providing a classification of the research results. An SMR can be helpful to researchers because it provides an overview of the literature at a given time for a specific topic of interest [12].

**Figure 2:**
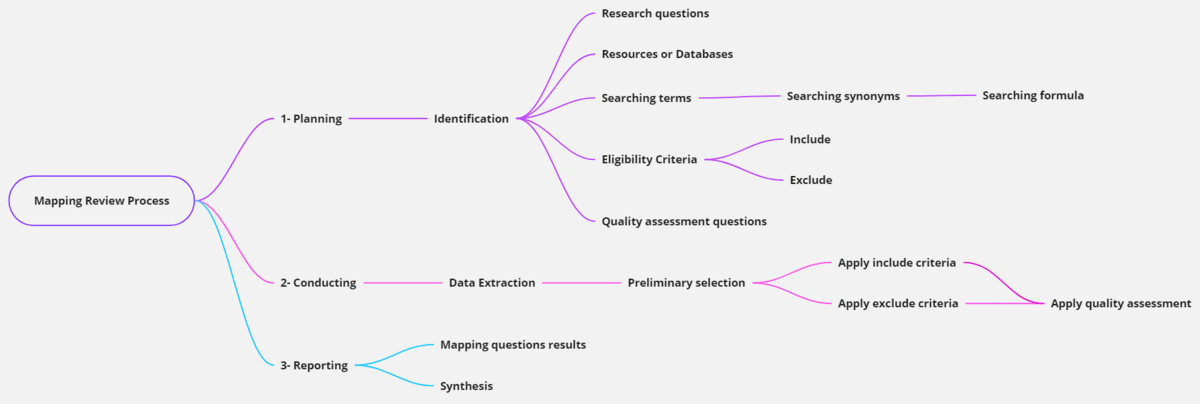
Mapping and review process for this Fast Healthcare Interoperability Resources study.

According to the study by Petersen et al [13], the mapping review process follows three steps: (1) planning the mapping, (2) conducting the mapping, and (3) reporting the results of the mapping. Furthermore, specific guidelines for a systematic mapping review in the software engineering domain as mentioned in the study by Brereton et al [14] suggest more detailed activities at each step (Figure 2):

- Planning: identification of the different items used for the research process, including RQs, resources and databases, search terms and formulas, inclusion and exclusion criteria, and questions for quality assessment (QA).
- Conducting: initial extraction of the studies was performed in this step. An initial preselection was performed, and both the inclusion and exclusion criteria were applied. The selected studies were then evaluated and filtered using QA questions.
- Reporting: during this step, mapping was conducted using the final selection of the studies, and a discussion of the results was presented.

### Research Questions

A set of 5 questions was defined (Table 3).

**Table 3:** Research questions.

RQ^a^ ID	RQ
RQ1	What are the main resources types studied or used in FHIR^b^ semantic interoperability studies?
RQ2	Which terminologies are used in FHIR semantic interoperability studies?
RQ3	What are the semantic research approaches most frequently applied to achieve semantic interoperability?
RQ4	What are the main types of contributions within the selected studies?
RQ5	What are the publication sources and types?

^a^RQ: research question.

^b^FHIR: Fast Healthcare Interoperability Resources.

### Mapping Search Strategy

#### Search String

The search string was established iteratively: first, by using “FHIR” as a search term in 3 databases and screening the results to look for more keywords dealing with the subject of interest, and second, by identifying additional terms and their synonyms based on the RQs.

The following is the search string composed using Boolean operators based on the identified terms: FHIR AND (Semantic OR terminology OR vocabulary OR Ontology OR mapping OR classification).

The Zotero reference management tool [15] was used to collect, organize, annotate, and review the abstract and full text to include or exclude studies that met the eligibility criteria. Zotero helps manage the large number of studies extracted for research.

#### Search Databases

The following 10 databases were used to identify and extract studies that satisfied the predefined search string: IEEE Xplore, ScienceDirect, Springer, Scopus, ACM, Web of Science, MEDLINE or PubMed, Compendex, Inspec, and Semantic Scholar.

#### Eligibility Criteria

Textbox 1 shows the inclusion and exclusion criteria for identified and extracted studies. Articles were excluded based on titles and abstracts as well as full-text reading in some cases.

**Inclusion and exclusion criteria for identified or extracted studies.**

**Inclusion criteria**

- Published between 2012 and 2022 (start date of 2012 was chosen because it was the date of publication of the first draft of the Fast Healthcare Interoperability Resources [FHIR]).
- Published in English or French

**Exclusion criteria**

- Studies not accessible in full text
- Books
- Studies reported on 2-3 pages
- Duplicate studies: We retained the most recent data
- Technical reports or summaries of conferences and editorials
- Studies that do not address semantics using FHIR
- Studies addressing enhancements to FHIR terminology and comparisons

### Quality Assessment

QA of a research publication is a crucial selection step to ensure that the selected studies have the appropriate impact for mapping reviews and further analysis [12]. For this purpose, we defined quality criteria scores based on the following questions.

- QA1: Are the objectives of the research clearly defined?
- QA2: Does the author present results that meet the stated research objectives?
- QA3: Is the method or technique used to achieve semantic interoperability clearly described?
- QA4: Is a literature review provided?
- QA5: Is the author’s contribution clearly stated?
- QA6: Is a result validation strategy presented?

Quality evaluation of the identified and extracted studies was performed by answering each QA question. The selected studies satisfied at least 4 of the 6 QA criteria. Others were rejected.

### Data Extraction and Data Synthesis

**Figure 3:**
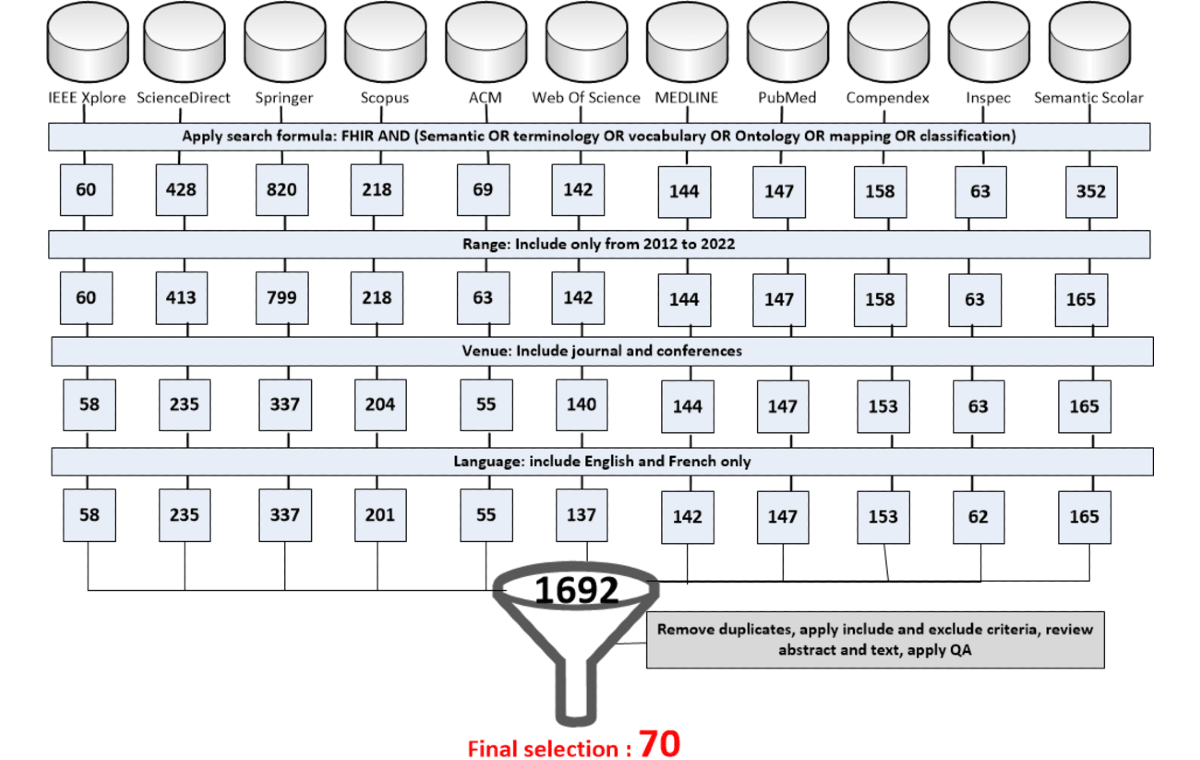
Search steps and results from databases. QA: quality assessment.

This SMR was conducted in 2022, with publications published from 2012 to 2022. A summary of the data extraction showing the selection filter effect is presented in Figure 3.

- 2601 results were obtained in the first step
- 2372 remained after applying the exclusion criteria for studies published out of the range of 2012‑2022
- 1701 remained after the applying the exclusion criteria on venue type is possible, when available
- 1692 for studies remained after applying the English or French language restriction
- 70 studies remained after removing duplicates, applying the additional inclusion, exclusion criteria and QA.

The 70 studies retained are listed in Multimedia Appendix 2 [16-85]. The extracted data are presented in Multimedia Appendix 3.

## Results

This section presents findings related to the 5 RQs listed in Table 3.

### RQ1: Main FHIR Resources Types Studied or Used

**Figure 4:**
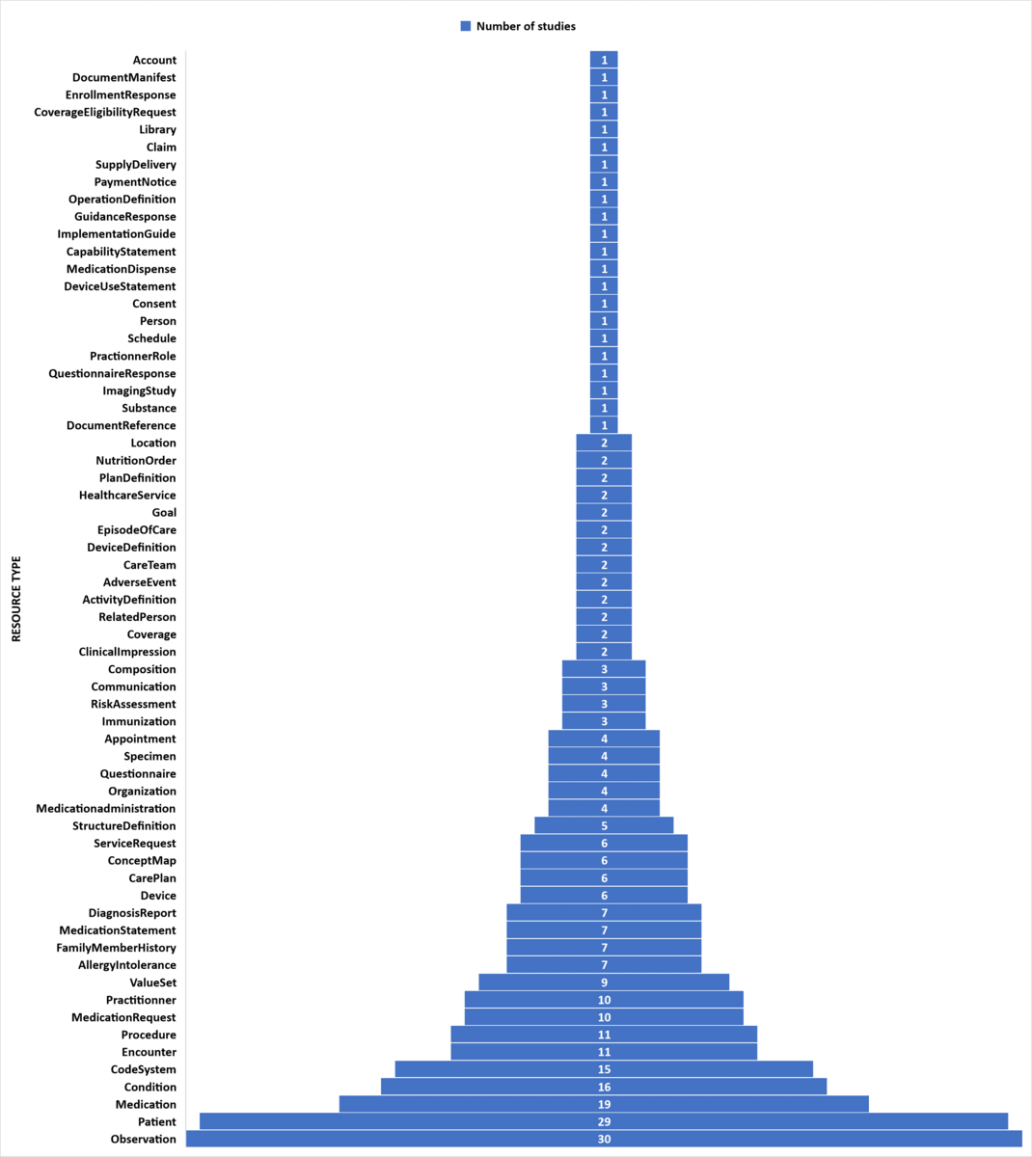
Distribution of the resources used.

We reviewed 70 selected studies and identified the types of FHIR resources that were used. Table 4 lists the 62 types of resources identified in the corresponding studies that have used them. In Figure 4, the resource types are presented, from most frequently used to least used.

The 10 most referred or used resource types in these studies were Observation (31/297, 10.4%), Patient (29/297, 9.8%), Medication (19/297, 6.4%), Condition (16/297, 5.4%), CodeSystem (15/297, 5.1%), Encounter (11/297, 3.7%), Procedure (11/297, 3.7%), Medication Request (10/297, 3.4%), Practitionner (10/297, 3.4%), and ValueSet (9/297, 3%).

The HL7 organization classifies the list of resources into five categories: (1) foundation, (2) base, (3) clinical, (4) financial, and (5) specialized resources. Each of these categories was further divided into 5 subcategories, except for the financial resources category, which was subdivided into 4 subcategories.

**Figure 5:**
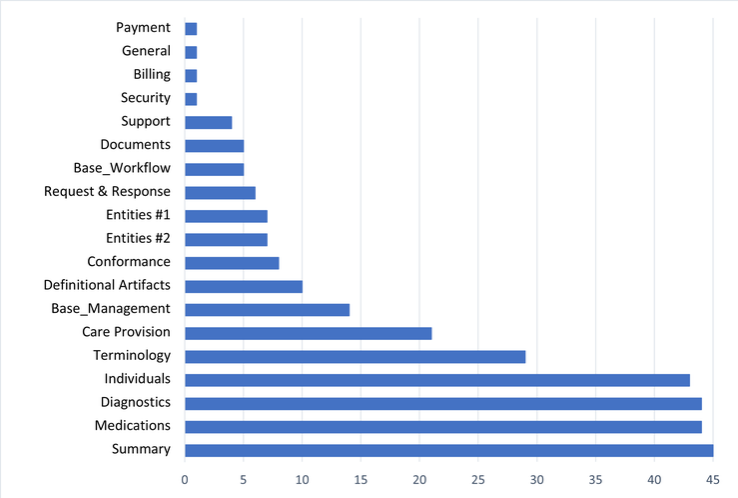
Distribution of the Fast Healthcare Interoperability Resources types used by category.

The resources inventoried in the 70 selected studies were mostly in 3 clinical subcategories: summary, medications, and diagnosis (Figure 5 and Multimedia Appendix 1). There is one exception to Patient and Encounter resources, which are in the base category and mainly used for identification purposes. We also noted the terminology subcategory (foundation category) among the top 10 most used resources. Terminology resources were used to map concepts to FHIR terminology (eg, SNOMED CT and LOINC).

**Table 4:** Fast Health Care Interoperability Resources types used and related studies.

Resource type	Study ID	Studies, n
Observation	S2, S42, S43, S68, S47, S60, S48, S6, S7, S8, S9, S11, S12, S40, S28, S13, S14, S15, S54, S55, S56, S38, S22, S61, S23, S62, S5, S34, S69, S70	30
Patient	S1, S42, S43, S68, S65, S46, S47, S60, S48, S50, S39, S9, S11, S13, S14, S15, S16, S54, S55, S30, S56, S38, S22, S23, S5, S34, S52, S69, S70	29
Medication	S1, S68, S36, S47, S63, S4, S60, S48, S6, S39, S9, S14, S54, S55, S31, S22, S61, S5, S69	19
Condition	S25, S68, S60, S6, S39, S8, S9, S11, S12, S40, S14, S15, S54, S22, S61, S62	16
CodeSystem	S37, S9, S29, S10, S40, S41, S17, S18, S20, S61, S33, S14, S15, S21, S28	15
Encounter	S1, S68, S60, S39, S9, S11, S16, S38, S23, S62, S5	11
Procedure	S68, S60, S39, S8, S9, S40, S61, S5, S62, S12, S69	11
Practitionner	S68, S47, S60, S50, S9, S15, S16, S34, S23, S70	10
MedicationRequest	S1, S68, S63, S4, S39, S53, S62, S22, S61, S69	10
ValueSet	S37, S9, S29, S40, S41, S17, S18, S61, S14	9
MedicationStatement	S68, S6, S8, S9, S40, S14, S61	7
FamilyMemberHistory	S68, S60, S39, S8, S15, S54, S61	7
AllergyIntolerance	S68, S60, S9, S67, S28, S15, S62	7
DiagnosisReport	S1, S40, S14, S5, S62, S34, S70	7
CarePlan	S68, S60, S50, S9, S23, S62	6
Device	S2, S43, S68, S60, S7, S39	6
ServiceRequest	S60, S39, S9, S38, S34, S69	6
ConceptMap	S37, S13, S41, S17, S19, S69	6
StructureDefinition	S44, S38, S53, S40, S69	5
Specimen	S60, S9, S40, S34	4
Questionnaire	S28, S15, S30, S23	4
Organization	S60, S16, S38, S23	4
MedicationAdministration	S47, S48, S39, S9	4
Appointment	S60, S9, S28, S69	4
Immunization	S60, S40, S62	3
Composition	S8, S61, S69	3
Communication	S39, S9, S69	3
RiskAssessment	S60, S28, S69	3
HealthcareService	S60, S9	2
Goal	S68, S62	2
EpisodeOfCare	S68, S23	2
DeviceDefinition	S2, S7	2
CareTeam	S68, S60	2
AdverseEvent	S68, S60	2
ActivityDefinition	S23, S43	2
RelatedPerson	S68, S60	2
Coverage	S9, S16	2
ClinicalImpression	S12, S23	2
Location	S68, S69	2
NutritionOrder	S68, S69	2
PlanDefinition	S23, S69	2
DocumentReference	S60	1
MedicationDispense	S39	1
DeviceUseStatement	S9	1
Consent	S9	1
Person	S28	1
Schedule	S15	1
PractionnerRole	S16	1
QuestionnaireResponse	S23	1
ImagingStudy	S23	1
Substance	S39	1
Account, Library, Claim, DocumentManifest, EnrollmentResponse, CoverageEligibilityRequest, SupplyDelivery, PaymentNotice, OperationDefinition, GuidanceResponse, ImplementationGuide, CapabilityStatement,	S69	1

### RQ2: Which FHIR Terminologies Were Used

A total of 9 distinct FHIR terminologies were used in the 70 selected studies (Table 5).

Their distribution and percentage are as follows:

- SNOMED CT (31/98, 32%) and LOINC (25/98, 26%) were the most commonly used.
- ICD-9 and ICD-10 (15/98, 15%) and Unified Medical Language System (13/98, 13%)
- RxNORM (9/98, 9%)
- The remaining terminologies (anatomical therapeutic chemical, quality data model, national drug file–reference terminology, and classification of instructional programs code) accounted for <10%.

**Table 5:** List of terminologies being used.

Terminology	Study ID
SNOMED CT^a^	S2, S34, S3, S35, S4, S48, S5, S49, S7, S8, S9, S29, S10, S11, S12, S40, S28, S53, S14, S16, S41, S31, S38, S18, S66, S22, S61, S23, S13, S25, S64
LOINC^b^	S2, S34, S3, S35, S5, S6, S7, S8, S27, S9, S11, S13, S16, S54, S30, S56, S41, S38, S20, S66, S21, S22, S23, S24, S36
ICD^c^-9 or ICD-10	S34, S1, S35, S49, S6, S9, S54, S30, S41, S17, S18, S66, S22, S33, S26
UMLS^d^	S34, S43, S37, S5, S27, S14, S19, S66, S21, S61, S64, S25, S8
RxNorm^e^	S36, S6, S8, S9, S14, S54, S31, S22, S61
ATC^f^	S1, S8
QDM^g^	S39
NDF-RT^h^	S48
CIP^i^ Code	S1

^a^SNOMED CT: Systematized Nomenclature of Medicine–clinical terms.

^b^LOINC: Logical Observation Identifiers Names and Codes.

^c^ICD: International Classification of Diseases.

^d^UMLS: Unified Medical Language System.

^e^RxNORM: medical prescription normalized Medical prescription.

^f^ATC: anatomical therapeutic chemical.

^g^QDM: quality data model.

^h^NDF-RT: national drug file–reference terminology.

^i^CIP: classification of instructional programs.

### RQ3: Semantic Research Approaches Most Frequently Applied

A total of six main semantic research approaches were identified from the solutions proposed in the 70 selected studies:

1. Mapping to FHIR terminology (mapping)
2. Terminology services (terminology)
3. Transformation, development, and validation resource description framework (RDF) or web ontology language (OWL)
4. Machine learning (ML) and natural language processing (NLP)
5. Semantic annotation (annotation)
6. Ontology-based approach (ontology)

Table 6 presents the semantic research approaches used to decrease the frequency of use in the selected studies.

**Table 6:** Semantic research approaches and related studies.

Semantic approach	Studies IDs	Distribution (N=126), n (%)
Mapping to Fast Healthcare Interoperability Resources terminologies (mapping)	S1, S2, S4, S5, S6, S7, S8, S9, S10, S11, S13, S14, S15, S16, S17, S18, S20, S21, S22, S23, S24, S25, S27, S28, S29, S30, S31, S32, S33, S38, S41	31 (24.6)
Terminology services (terminology)	S5, S13, S17, S18, S19, S20, S21, S28, S30, S33, S34, S35, S36, S37, S38, S39, S40, S41	18 (14.3)
Transformation or development or validation resource description framework or web ontology language	S3, S10, S12, S14, S35, S42, S43, S44, S45, S46, S47, S48, S49, S50, S51, S52, S53, S54, S55, S56, S57, S58, S59, S69	24 (19)
Semantic annotation (annotation)	S2, S3, S6, S7, S14, S25, S27, S30, S31, S32, S43, S45, S48, S60, S61, S62, S63, S64	18 (14.3)
Machine learning or natural language processing	S5, S6, S8, S14, S25, S26, S31, S32, S43, S46, S49, S54, S60, S61, S62, S63, S64, S65, S66, S67	20 (15.9)
Ontology-based approach (ontology)	S3, S11, S12, S13, S26, S40, S44, S46, S47, S49, S50, S55, S66, S68, S70	15 (11.9)

The distribution of the 6 semantic approaches is as follow: mapping (31/126, 24.6%), RDF or OWL (24/126, 19%), ML and NLP (20/126, 15.9%), annotation (18/126, 14.3%), terminology (18/126, 14.3%), and ontology (15/126, 11.9%).

The selected studies used at least one of the 6 identified semantic research approaches, and most studies used more than one research approach. For example, 22 of the 31 studies listed in Table 6 used mapping and other approaches (70%), whereas only 29% (9/31) used mapping alone.

To better understand the frequency of combinations of more than one semantic research approach, we identified a number of studies using more than one approach. We performed the same exercise for RDF or OWL followed by annotation. Table 7 shows that most combined semantic approaches are annotation and ML and NLP, mapping and terminology services, mapping and annotation, RDF or OWL, and ontology.

**Table 7:** Distribution of the combined approaches

Category	Mapping	Terminology	RDF^a^ or OWL^b^	Annotation	ML^c^ and NLP^d^	Ontology
Mapping	—^e^	11	2	9	7	2
Terminology	—	—	0	1	1	2
RDF and OWL	—	—	—	5	5	8
Annotation	—	—	—	—	11	1
ML and NLP	—	—	—	—	—	4
Ontology	—	—	—	—	—	—

^a^RDF: resource description framework.

^b^OWL: web ontology language.

^c^ML: machine learning.

^d^NLP: natural language processing.

^e^Not applicable.

**Figure 6:**
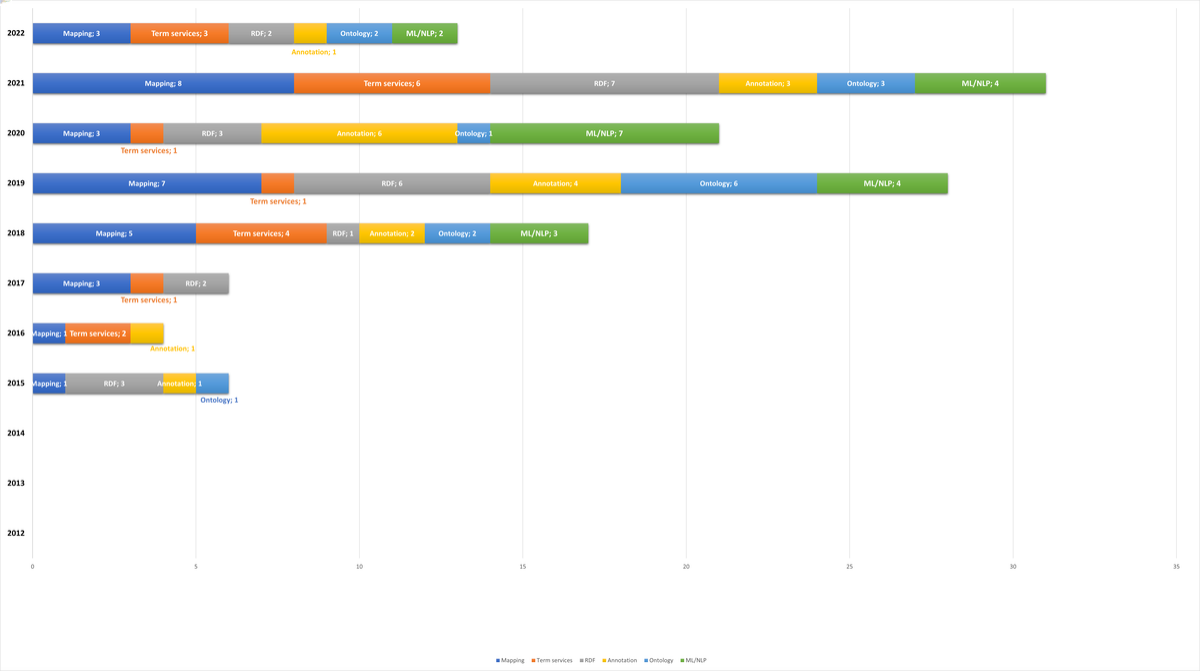
Timeline of semantic research approaches used in the selected studies. ML: machine learning; NLP: natural language processing; RDF: resource description framework.

The next analysis related to the proposed approach examined how these approaches evolved over time. Figure 6 shows how these approaches were used over time. The main finding are (1) ML and NLP-based proposals emerged in 2018 in FHIR semantic interoperability studies, (2) the other approaches span the full period, and (3) no specific research approach dominates the FHIR semantic interoperability research field.

### RQ4: Main Types of Contributions

The objective of RQ4 was to identify the contribution types within each of the 70 selected studies. For this purpose, five main contributions were identified: (1) new framework or architecture intended to enable semantic interoperability (framework or architecture proposal), (2) evaluation or comparison of different techniques (eg, comparison of ontologies), (3) new ontology, model or enhancement of an existing one (model proposal), (4) a new technique or improvement of an existing technique (new technique proposal), and (5) a tool proposal through the development of a new tool or an enhancement to existing ones to facilitate achieving semantic interoperability (tool proposal).

Table 8 presents the distribution of the 70 studies selected according to contribution type.

**Figure 7:**
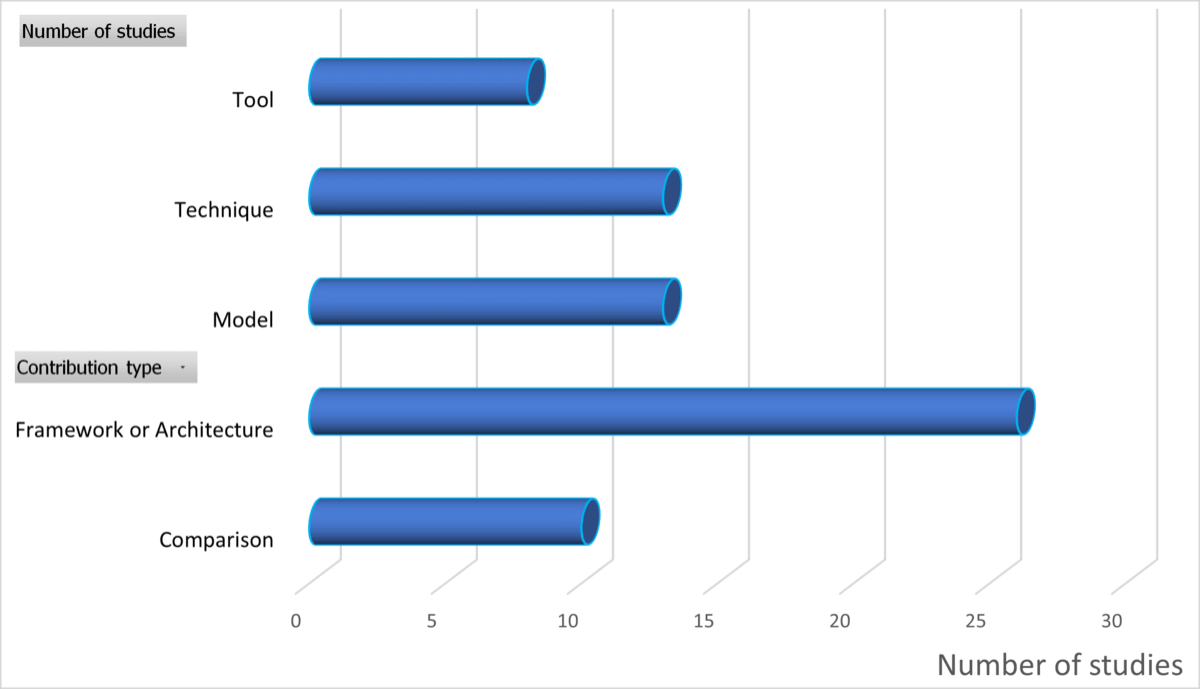
Distribution of the studies by contribution type.

Figure 7 shows the distribution of studies by contribution type.

The most frequent contribution type was the proposal of a framework or architecture (26/70, 37%), followed by model proposals (13/70, 19%), new or improved techniques (13/70, 19%), comparison services (10/70, 14%), and tool proposals (8/70, 11%).

**Table 8:** Studies by contribution type.

Contribution type	Studies
Framework or architecture	S2, S5, S7, S8, S14, S16, S20, S22, S23, S25, S26, S29, S31, S34, S35, S38, S41, S43, S51, S53, S54, S61, S62, S65, S66, S68
Comparison	S4, S15, S17, S32, S33, S46, S56, S58, S59, S67
Model	S1, S3, S6, S30, S39, S40, S45, S48, S49, S50, S55, S60, S63
Technique	S9, S10, S12, S18, S19, S21, S24, S27, S42, S47, S64, S69, S70
Tool	S11, S13, S28, S36, S37, S44, S52, S57

### RQ5: Publications Sources and Types

#### Publication Sources

**Figure 8:**
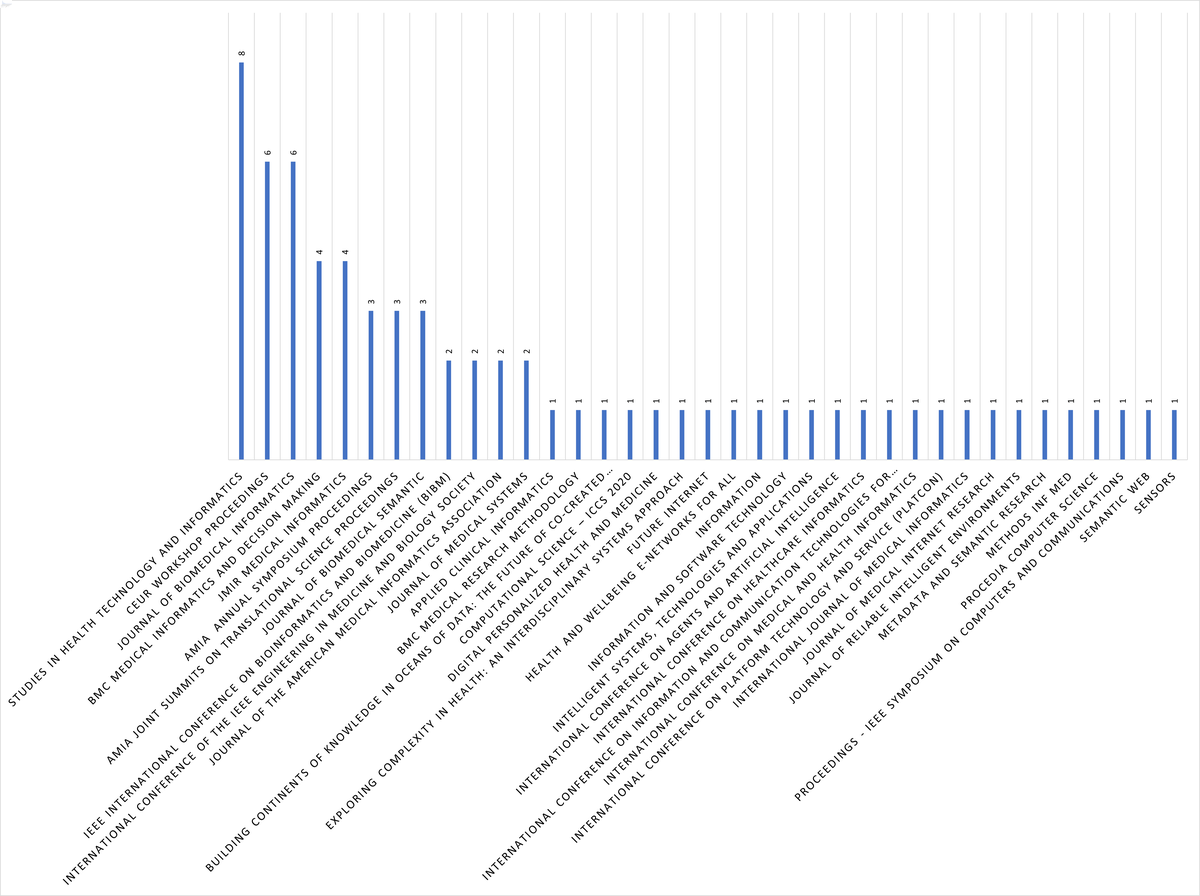
Publication sources and corresponding number of studies.

The distribution of publication sources and the number of studies is summarized as follows (Figure 8):

- The top publication sources were *Studies in Health Technology and Informatics* (8/70, 11%), followed by *CEUR Workshop* and the *Journal of Biomedical Informatics* (6/70, 9%).
- *JMIR Medical Informatics* and *BMC Medical Informatics and Decision Making* follow with (4/70, 6%).
- Approximately (3/70, 4%) of the publications are from 3 sources: *Journal of Biomedical Semantic*, American Medical Informatics Association (AMIA) Joint Summits on Translational Science proceedings, and AMIA Annual Symposium.
- Approximately (2/70, 3%) of the publications are from 4 sources: the *Journal of Medical Systems* , IEEE International Conference on Bioinformatics and Biomedicine, International Conference of the IEEE Engineering in Medicine and Biology Society, and the *Journal of the AMIA*.
- Each of the remaining 25 publications represented 1%, with 1 study per source.

#### Publication Types

The distribution of the selected studies by venue type (Table 9) showed that most of selected studies were published in a journal (46/70, 66%), followed by conferences (15/70, 21%), workshops (6/70, 9%), and symposiums (3/70, 4%).

**Table 9:** Number of studies per venue type (N=70).

Venue type	Studies, n (%)
Journal	46 (66)
Conference	15 (21)
Workshop	6 (9)
Symposium	3 (4)

## Discussion

### Overview

This section presents a discussion of each of the key findings of the RQs.

1. Most studies have concentrated on clinical data resources; therefore, the focus has been on patient summaries.
2. Terminology mapping to SNOMED CT and LOINC was performed in more than half of the selected studies. None of the 5 approaches was dominant in the proposed solutions to semantic interoperability challenges.
3. Most of the selected studies proposed a framework or architecture as a solution to enable semantic interoperability.
4. In recent years, research on semantic interoperability has increased since 2018.

### RQ Results

#### RQ1: What Are the Main Resource Types Studied or Used?

As most studies concentrated on clinical data resources (eg, Observation, Medication, Condition, Procedure, and MedicationRequesL), the focus has been on patient summary including the medical condition, medication, and diagnosis.

The attributes of these resource-use codes (CodeableConcept) and terminology standards, such as SNOMED CT and LOINC, are already included in the FHIR body. For example, the Observation resource has an attribute code in which the practitioner or user can document the observation type using LOINC code. Similarly, the Medication resource prescribed can be entered either as a code using SNOMED CT or as a textual description.

In contrast, other types of resources (eg, BodyOfStructure) were not addressed in the selected studies.

#### RQ2: Which Terminologies Were Used?

Terminology mapping to SNOMED CT and LOINC was present in more than half of the selected studies, where SNOMED CT is used mainly for medication, procedure, and condition (problem); SNOMED CT or ICD-10 for diseases and diagnosis; and LOINC for observation and diagnostic report.

Some countries have attempted to develop a consensus on the terminology to be used at the regional or national level. For example, SNOMED CT was adopted as a single terminology in the United Kingdom by the National Health Service (S12). In Switzerland, the federal initiative of the Swiss Personalized Health Network created a semantic framework available for health communities aiming to use the existing standards instead of building a new one, and the semantics were mapped by domain experts on the existing standards, such as SNOMED CT, LOINC, and ICD. RDF is used to store and transport data. In another regional initiative in Italy, the LOINC vocabulary was used to represent concepts already defined in local and standardized terminologies (S20).

#### RQ3: What Are the Most Frequently Applied Research Approaches to Achieve Semantic Interoperability?

None of the 5 approaches were dominant in the proposed solutions to semantic interoperability challenges. The emerging ML and NLP approach is mostly applied to annotation.

ML and NLP techniques, which have achieved significant penetration in many other information system or information technology domains, have also been used in FHIR semantic interoperability research. On the one hand, the capability of the ML and NLP techniques for automation and prediction is often required for complex use cases. On the other hand, RDF representation has the specific advantage of containing information about semantic links and dependencies between multiple resources. This could explain why transformation to or from RDF is useful for semantic interoperability.

#### RQ4: What Are the Main Types of Contributions Within the Selected Studies?

Most of the selected studies proposed a framework or architecture as a solution to enable semantic interoperability. Research on achieving semantic interoperability using FHIR has tackled different steps and activities: collecting, extracting, and annotating data; modeling data from legacy systems; and applying transformation and mapping to FHIR models and terminologies. The following research topics were identified.

- One of the main topics in the selected studies was related to unstructured data because valuable information can be extracted from free text for research and health analytics. The standardization of unstructured clinical notes was the focus of the research for specific use cases in the selected studies. The process of concept extraction and mapping text to standardized terminologies or ontologies requires more automation.
- Research on unstructured data using annotation and NLP is a relatively new field for the FHIR community, and challenges related to protecting privacy and security (deidentification and pseudonymization) remain open questions.
- The manual mapping of clinical terminology requires significant effort. The use of a tool can reduce the effort required for mapping and increase the adoption of terminology.
- Another challenge is related to languages other than English. In S24, a tool was developed to map Russian local terminology to LOINC.
- Even though the FHIR standard allows the use of clinical terminologies such as SNOMED CT and LOINC, users still enter information as text or use local terminologies. For example, selecting an appropriate concept from SNOMED CT is not a trivial task. To this end, a proposed solution called Ontoserver (eg, a clinical terminology service) offers a prefix-based search algorithm to help users easily find content and enter coded data. Ontoserver was used in several studies (S5, S10, S11, S17, S18, S40, and S41), and limitations related to its performance were reported.
- Comparison and mapping ontologies: detecting similarities and differences is still of interest and may attract more researchers.

#### RQ5: What Are the Publication Sources and Types?

**Figure 9:**
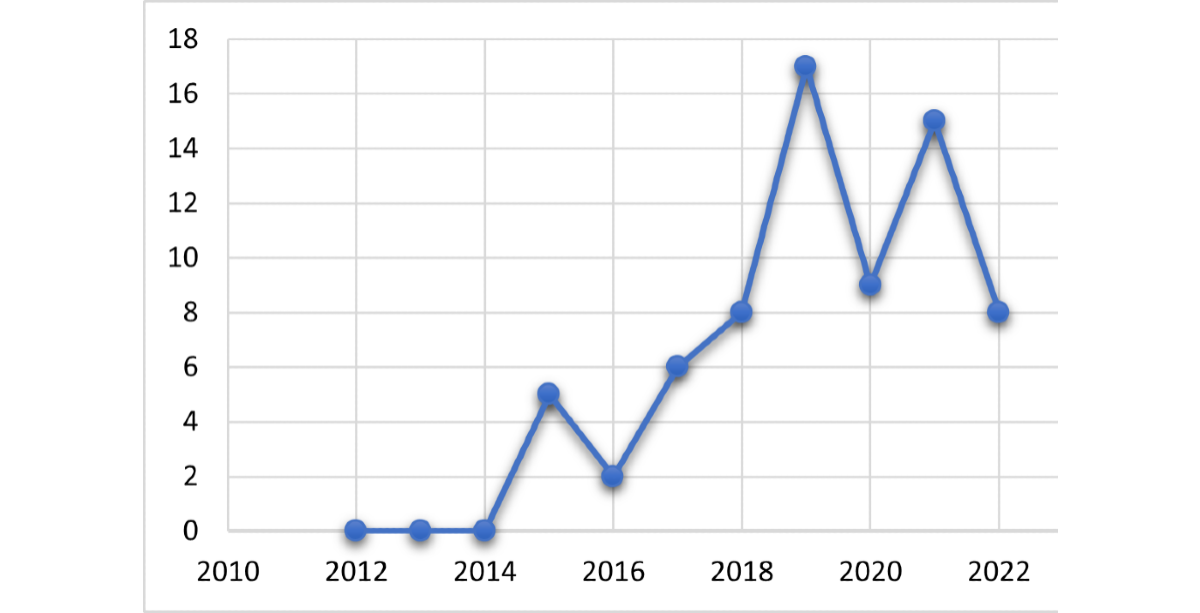
Number of studies over time.

Research on FHIR semantic interoperability emerged in 2015 and has increased since 2018. This probably is linked to the first release HL7 FHIR with a “Normative” status in 2018 indicating that its content has been recognized at the international level as stable for use and has been “locked” for several years. The publication trend in Figure 9 that shows the distribution of the selected studies over time confirms that research on this topic is still active.

### Assumptions and Limitations

This study was limited to semantic studies using only the FHIR standard, and it did not search for other studies related to semantic interoperability in general; therefore, this SMR focused exclusively on studies addressing FHIR implementation.

Furthermore, only studies from the 10 database sources specified were considered, and commercial solutions were excluded.

During the classification of the contribution based on 5 categories, 2 studies (S40 and S49) were considered in 2 different categories (model and technique). Both situations were evaluated and the results were only slightly different.

### Conclusions

This SMR inventoried and analyzed the findings on semantic interoperability using FHIR over the last decade (2012-2022) in 70 selected studies from 10 databases. It presents a classification of the selected studies according to the semantic approaches used and their contributions. It also provides the frequency of use of FHIR resources and terminologies.

These findings provide researchers with an inventory of the approaches used to achieve semantic interoperability using the FHIR. The SMR also documented the frequency with which the proposed approaches were used. This may help identify avenues for research on semantic interoperability. It can also be used as a guideline by the FHIR community for the future development of approaches and types of resources that are not well covered in research published to date.

Indeed, understanding and interpreting the exchanged data in the EHR ecosystem needs to be supported by further research using, for instance, FHIR in mapping, RDF or OWL, and comparing ontologies and terminology services.

Semantic interoperability is still an active research field, and there is no broad consensus in the health care community on how to achieve full semantic interoperability between information systems. The main issues reported are related to the difficulty associated with the use of different terminologies and the efforts required to successfully map to FHIR terminology. Even worse, when no terminology is used, unstructured data would still need to be extracted and mapped to the appropriate concept code for FHIR terminology.

This SMR provides a classification of the different solutions proposed to address semantic interoperability using FHIR at different steps: collecting, extracting and annotating data, modeling EHR data from legacy systems, and applying transformation and mapping to FHIR models and terminologies. The use of ML and NLP for unstructured data is promising and has been applied to specific use case scenarios. In addition, terminology services are needed to accelerate their use and adoption; furthermore, techniques and tools to automate annotation and ontology comparison should help reduce human interaction.

Future work will be devoted to the FHIR-based framework for the semantic interoperability of EHR data.

## Appendix

Multimedia Appendix 1 Targeted resources from a semantic point of view.

Multimedia Appendix 2 List of the selected studies (N=70).

Multimedia Appendix 3 Extracted data and searching strategy results.

## Abbreviations

AMIA: American Medical Informatics Association
EHR: electronic health record
FHIR: Fast Healthcare Interoperability Resources
HL7: Health Level Seven International
ICD: International Classification of Diseases
IEEE: Institute of Electrical and Electronics Engineers
LOINC: Logical Observation Identifiers Names and Codes
ML: machine learning
NLP: natural language processing
OWL: web ontology language
QA: quality assessment
RDF: resource description framework
RQ: research question
SMR: systematic mapping review
SNOMED CT: Systematized Nomenclature of Medicine–clinical terms
