# Supplementary figures and images for: Electronic Health Record and Semantic Issues Using Fast Healthcare Interoperability Resources: Systematic Mapping Review
Source: J Med Internet Res. 2024 Jan 30;26:e45209. doi: 10.2196/45209 (PMC10865191; doi:10.2196/45209)

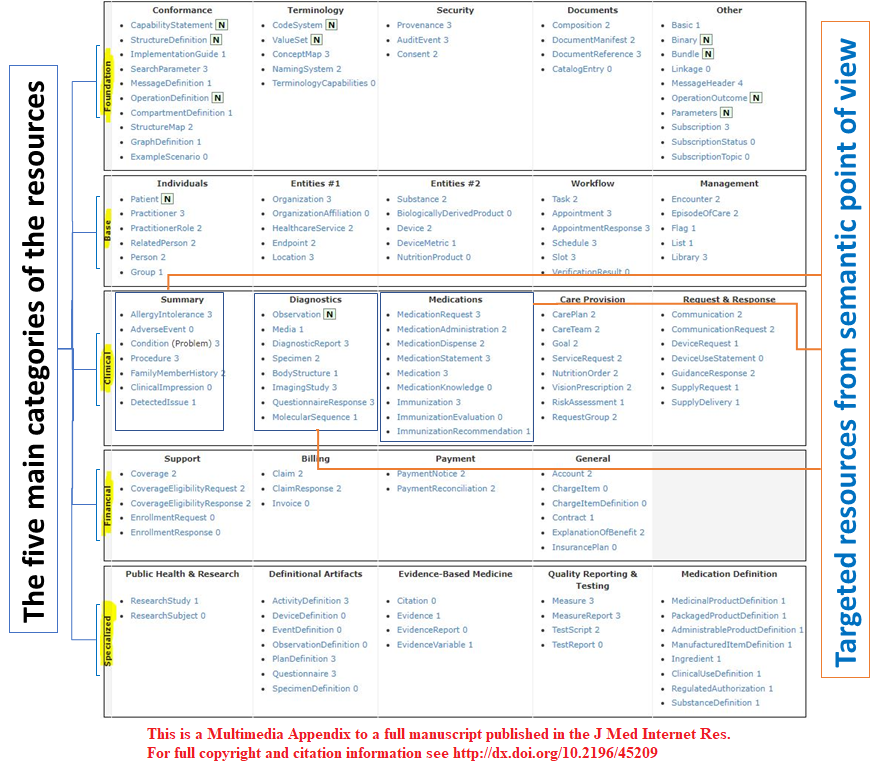

Supplement: Multimedia Appendix 1 [file jmir_v26i1e45209_app1.png]
