# Supplementary material for: Electronic Health Record and Semantic Issues Using Fast Healthcare Interoperability Resources: Systematic Mapping Review
Source: J Med Internet Res. 2024 Jan 30;26:e45209. doi: 10.2196/45209 (PMC10865191; doi:10.2196/45209)
Supplement: Multimedia Appendix 2 [file jmir_v26i1e45209_app2.pdf]

Appendix 1: List of the Selected Studies (N = 70)

ID	Title	Authors	Year	Venue type	Reference
S1	A Fast Healthcare Interoperability Resources (FHIR) layer implemented over i2b2	Abdelali Boussadi, Eric Zapletal	2017	Journal	[16]
S2	A FHIR based architecture of a multiprotocol IoT Home Gateway supporting dynamic plug of new devices within instrumented environments	Paolo Zampognaro, Giovanni Paragliola, Vincenzo Falanga	2021	Conference	[17]
S3	A sustainable HL7 FHIR based ontology for PHR data	Vassilis Kilintzis, Alexandra Kosvyra, Nikolaos Beredimas, Pantelis Natsiavas, Nicos Maglaveras, Ioanna Chouvarda	2019	Conference	[18]
S4	An Empirical Approach to Enhancing Terminology Binding - An HL7 FHIR SNOMED CT Example	Kirstine Rosenbeck Gøeg, Mark Hummeluhr	2018	Conference	[19]
S5	APERITIF - Automatic Patient Recruiting for Clinical Trials Based on HL7 FHIR	Alexandra Banacha, Hannes Ulrich, Björn Kroll, Alexander Kiel, Josef Ingenerf, Ann-Kristin Kock-Schoppenhauer	2021	Journal	[20]
S6	Clinical Text Mining on FHIR	Philipp Daumke, Kai U. Heitmann, Simone Heckmannb, Catalina Martínez-Costa, Stefan Schulz	2019	Journal	[21]
S7	Definition of an FHIR-based multiprotocol IoT home gateway to support the dynamic plug of new devices within instrumented environments	Paolo Zampognaro, Giovanni Paragliola, Vincenzo Falanga	2021	Journal	[22]
S8	Developing a FHIR-based EHR phenotyping framework: A case study for identification of patients with obesity and multiple comorbidities from discharge summaries	Na Hong, Andrew Wen, Daniel J. Stone, Shintaro Tsuji, Paul R. Kingsbury, Luke V. Rasmussen, Jennifer A. Pacheco, Prakash Adekkanattu, Fei Wang, Yuan Luo, Jyotishman Pathak, Hongfang Liu, Guoqian Jiang	2019	Journal	[23]
S9	Enhancing narrative clinical guidance with computer-readable artifacts: Authoring FHIR implementation guides based on WHO recommendations	Jennifer Shivers, Joseph Amlung, Natschja Ratanaprayul, Bryn Rhodes, Paul Biondich	2021	Journal	[24]
S10	FHIR OWL: Transforming OWL ontologies into FHIR terminology resources	Alejandro Metke-Jimenez, Michael Lawley, David Hansen	2019	Symposium	[25]
S11	FHIRCap: Transforming REDCap forms into FHIR resources	Alejandro Metke-Jimenez, David Hansen	2019	Journal	[26]
S12	From SNOMED CT expressions to an FHIR RDF representation: Exploring the benefits of an ontology-based approach	Mercedes Arguello-Casteleiro, Catalina Martínez-Costa, Julio Des-Diz, Nava Maroto, Maria Jesus Fernandez-Prieto	2019	Workshop	[27]
S13	i2b2 implemented over SMART-on-FHIR	Nicolas Paris, Michael Mendis, Christel Daniel, Shawn Murphy, Xavier Tannier, Pierre Zweigenbaum	2018	Journal	[28]

S14	Integrating Structured and Unstructured EHR Data Using an FHIR-based Type System: A Case Study with Medication Data	Na Hong, Andrew Wen, Feichen Shen, Sunghwan Sohn, Sijia Liu, Hongfang Liu, Guoqian Jiang	2018	Journal	[29]
S15	Investigation of Content Overlap in Proprietary Medical Mappings	Yulia Lenivtseva, Georgy Kopanitsa	2019	Journal	[30]
S16	Lessons Learned in Creating Interoperable Fast Healthcare Interoperability Resources Profiles for Large-Scale Public Health Programs	Susan A. Matney, Bret Heale, Steve Hasley, Emily Decker, Brittni Frederiksen, Nathan Davis, Patrick Langford, Nadia Ramey, Stanley M. Huff	2019	Journal	[31]
S17	Perspectives and Obstacles for Transforming Terminologies into FHIR CodeSystems Exemplified by Alpha-ID	Abdul-Mateen Rajput, Cora Drenkhahn	2021	Journal	[32]
S18	Providing ART-DECOR valuesets via FHIR terminology servers -a technical report	Joshua Wiedekopf, Cora Drenkhahn, Hannes Ulrich, Ann-Kristin Kock-Schoppenhauer, Josef Ingenerf	2021	Journal	[33]
S19	Representing UMLS knowledge using FHIR Terminological Resources	Rishi Saripalle	2019	Conference	[34]
S20	Semantics Management for a Regional Health Information System in Italy by CTS2 and FHIR	Roberta Gazzarata, Norbert Maggib, Luca Douglas Magnoni, Maria Eugenia Monteverde, Carmelina Ruggiero, Mauro Giacomini	2021	Journal	[35]
S21	Semi-Automated Approach to Validate and Enrich LOINC Codes by FHIR Server	Abdul Mateen Rajput, Ana Grönke, Wibke Johannis	2021	Conference	[36]
S22	SMART-on-FHIR implemented over i2b2	Kavishwar B Wagholikar, Joshua C Mandel, Jeffery G Klann, Nich Wattanasin, Michael Mendis, Christopher G Chute, Kenneth D Mandl, Shawn N Murphy	2017	Journal	[37]
S23	Towards achieving semantic interoperability of clinical study data with FHIR	Hugo Leroux, Alejandro Metke-Jimenez, Michael J. Lawley	2017	Journal	[38]
S24	Using Semi-automated Approach for Mapping Local Russian Laboratory Terms to LOINC	Georgy Kopanitsa, Maxim Taranik	2015	Journal	[39]
S25	A corpus-driven standardization framework for encoding clinical problems with HL7 FHIR	Kevin J. Peterson, Guoqian Jiang, Hongfang Liu	2020	Journal	[40]
S26	A Semantic Technologies Toolkit for Bridging Early Diagnosis and Treatment in Brain Diseases: Report from the Ongoing EU-Funded Research Project ALAMEDA	Christoniki Maga-Nteve, Efstratios Kontopoulos, Nikos Tsolakis, Ioannis Katakis, Evangelos Mathioudis, Panagiotis Mitzias, Konstantinos Avgerinakis, Georgios Meditskos, Anastasios Karakostas Stefanos Vrochidis, Ioannis Kompatsiaris	2022	Conference	[41]
S27	ELaPro, a LOINC-mapped core dataset for top laboratory procedures of eligibility screening for clinical trials	Ahmed Rafee, Sarah Riepenhausen, Philipp	2022	Journal	[42]

		Neuhaus, Alexandra Meidt, Martin Dugas, Julian Varghese			
S28	HL7 FHIR with SNOMED-CT to Achieve Semantic and Structural Interoperability in Personal Health Data: A Proof-of-Concept Study	Ayan Chatterjee, Nibedita Pahari, Andreas Prinz	2022	Journal	[43]
S29	Fast Healthcare Interoperability Resources, Clinical Quality Language, and Systematized Nomenclature of Medicine—Clinical Terms in Representing Clinical Evidence Logic Statements for the Use of Imaging Procedures: Descriptive Study	Eseosa Odigie, Ronilda Lacson, Ali Raja, David Osterbur, Louise Schneider, Ramin Khorasani	2019	Journal	[44]
S30	Metadata Repository for Improved Data Sharing and Reuse Based on HL7 FHIR	Hannes Ulrich, Ann-Kristin Kock, Petra Duhm-Harbeck, Jens K. Habermann, Josef Ingener	2016	Conference	[45]
S31	Opioid2FHIR: A system for extracting FHIR-compatible opioid prescriptions from clinical text	Jingqi Wang, William Christopher Mathews, Huy Anh Pham, Hua Xu, Yaoyun Zhang	2020	Conference	[46]
S32	Ordinal labels in machine learning: a user-centered approach to improve data validity in medical settings	Andrea Seveso, Andrea Campagner, Davide Ciucci, Federico Cabitza	2020	Journal	[47]
S33	TerminoDiff – Detecting Semantic Differences in HL7 FHIR CodeSystems	Joshua Wiedekopf, Cora Drenkhahn, Lorenz Rosenau, Hannes Ulrich, Ann-Kristin Kock-Schoppenhauer, Josef Ingenerf	2022	Journal	[48]
S34	A FHIR-based System for the Generation and Retrieval of Clinical Documents	Crescenzo Diomaiuta, Mario Sicuranza, Mario Ciampi, Giuseppe De Pietro	2017	Conference	[49]
S35	A National, Semantic-Driven, Three-Pillar Strategy to Enable Health Data Secondary Usage Interoperability for Research Within the Swiss Personalized Health Network: Methodological Study	Christophe Gaudet-Blavignac, Jean Louis Raisaro, Vasundratouré, Sabine Österle, Katrin Crameri, Christian Lovis	2021	Journal	[50]
S36	A Pharmacogenomics Clinical Decision Support Service Based on FHIR and CDS Hooks	R.H. Dolin, A.Boxwal, J. Shalaby	2018	Journal	[51]
S37	An Interoperable UMLS Terminology Service Using FHIR	Rishi Saripalle, Mehdi Sookhak, Mahboobeh Haghparast	2020	Journal	[52]
S38	Patients Decision Aid System Based on FHIR Profiles	Ilia Semenov, Georgy Kopanitsa, Dmitry Denisov, Yakovenko Alexandr, Roman Osenev, Yury Andreychuk	2018	Journal	[53]
S39	Developing a data element repository to support EHR-driven phenotype algorithm authoring and execution	Guoqian Jiang A, Richard C. Kiefer, Luke V. Rasmussen, Harold R. Solbrig, Huan Mo, Jennifer A. Pacheco, Jie Xu, Enid Montague, William K. Thompson, Joshua C. Denny, Christopher G. Chute, Jyotishman Pathak	2016	Journal	[54]

S40	Generation of a Fast Healthcare Interoperability Resources (FHIR)-based Ontology for Federated Feasibility Queries in the Context of COVID-19: Feasibility Study	Lorenz Rosenau; Raphael W Majeed, Josef Ingenerf1; Alexander Kiel, Björn Kroll; Thomas Köhler; Hans-Ulrich Prokosch; Julian Gruendner	2022	Journal	[55]
S41	Ontoserver: a syndicated terminology server	Alejandro Metke-Jimenez, Jim Steel, David Hansen, Michael Lawley	2018	Journal	[56]
S42	A FHIR-to-RDF converter	Gerhard Kober, Adrian Paschke	2019	Workshop	[57]
S43	A Flexible Semantic Integration Framework for Fully-integrated EHR based on FHIR Standard	Ahmed Dridi, Salma Sassi, Richard Chbeir, Sami Faiz	2020	Conference	[58]
S44	A Methodology for an Auto-Generated and Auto-Maintained HL7 FHIR OWL Ontology for Health Data Management	Vassilis Kilintzis, Vasileios C. Alexandropoulos, Nikolaos Beredimas, Nicos Maglaverasa	2021	Journal	[59]
S45	A reusable ontology for primitive and complex HL7 FHIR data types	Nikolaos Beredimas, Vassilis Kilintzis, Ioanna Chouvarda,	2015	Conference	[60]
S46	A Semantic Similarity Evaluation for Healthcare Ontologies Matching to HL7 FHIR Resources	Athanasios Kiourtis, Argyro Mavrogiorgou, Dimosthenis Kyriazisa	2020	Conference	[61]
S47	Aggregating the syntactic and semantic similarity of healthcare data towards their transformation to HL7 FHIR through ontology matching	Athanasios Kiourtis, Sokratis Nifakos, Argyro Mavrogiorgou, Dimosthenis Kyriazis	2019	Journal	[62]
S48	Annotating FHIR-RDF-graphs with medication knowledge	Gerhard Kober	2020	Workshop	[63]
S49	Automatic Stroke Medical Ontology Augmentation with Standard Medical Terminology and Unstructured Textual Medical Knowledge	Soonhyun Kwon, Jaehak Yu, Sejin Park, Jong-Arm Jun, Cheol-Sig Pyo	2021	Conference	[64]
S50	COC: An Ontology for Capturing Semantics of Circle of Care	Xiao Dong, Reza Samavi, Thodoros Topaloglou	2015	Journal	[65]
S51	Developing A Semantic Web-based Framework for Executing the Clinical Quality Language Using FHIR	Guoqian Jiang, Eric Prud'hommeaux, Guohui Xiao, Harold R. Solbrig	2017	Workshop	[66]
S52	Development of a FHIR RDF data transformation and validation framework and its evaluation	Eric Prud'hommeaux, Josh Collins, David Booth, Kevin J. Peterson, Harold R. Solbrig, Guoqian Jiang	2021	Journal	[67]
S53	Implementing a New FHIR RDF Specification for Semantic Clinical Data Using a JSONLD- based Approach	Deepak K. Sharma, Eric Prud'hommeaux, David Booth, Kevin J. Peterson1, Daniel J. Stone1, Harold Solbrig, Guohui Xiao, Emily Pfaff, Guoqian Jiang	2022	Workshop	[68]
S54	Leveraging Genetic Reports and Electronic Health Records for the Prediction of Primary Cancers: Algorithm Development and Validation Study	Nansu Zong, Victoria Ngo, Daniel J Stone, Andrew Wen, Yiqing Zhao, Yue Yu, Sijia Liu, Ming Huang, Chen Wang, Guoqian Jiang	2021	Journal	[69]
S55	Medical information-graphs, based on ontologies and FHIR	Gerhard Kober, Adrian Paschke	2021	Workshop	[70]
S56	Modeling Medical Guidelines by Prova and SHACL Accessing FHIR/RDF. Use Case: The Medical ABCDE Approach.	Gerhard Kober, Livio Robaldo, Adrian Paschke	2022	Journal	[71]

S57	Providing Full Semantic Interoperability for the Fast Healthcare Interoperability Resources Schemas with Resource Description Framework	Maria Penna Luz, Joyce Rocha De Matos Nogueira	2015	Conference	[72]
S58	ShExMap and IPSM-AF—Comparison of RDF Transformation Technologies	Paweł Szmeja, Eric Prud'hommeaux	2021	Journal	[73]
S59	Using Shape Expressions (ShEx) to Share RDF Data Models and to Guide Curation with Rigorous Validation	Katherine Thornton, Harold Solbrig, Gregory S. Stupp, Jose Emilio Labra Gayo, Daniel Mietchen, Eric Prud'hommeaux, And Andra Waagmeester	2019	Journal	[74]
S60	Analyzing Patient Secure Messages Using a Fast Health Care Interoperability Resources (FIHR)-Based Data Model: Development and Topic Modeling Study	Amrita De, Ming Huang, Tinghao Feng, Xiaomeng Yue, Lixia Yao	2021	Journal	[75]
S61	Standardizing Heterogeneous Annotation Corpora Using HL7 FHIR for Facilitating their Reuse and Integration in Clinical NLP	Na Hong, Andrew Wen, Majid Rastegar Mojarad, Sunghwan Sohn, Hongfang Liu, Guoqian Jiang	2018	Symposium	[76]
S62	Using FHIR to Construct a Corpus of Clinical Questions Annotated with Logical Forms and Answers	Sarvesh Soni, Meghana Gudala, Daisy Zhe Wang, Kirk Roberts	2019	Symposium	[77]
S63	An annotation and modeling schema for prescription regimens	John Aberdeen, Samuel Bayer, Cheryl Clark, Meredith Keybl And David Tresner-Kirsch	2019	Journal	[78]
S64	Annotation and extraction of age and temporally-related events from clinical histories	Judy Hong, Anahita Davoudi, Shun Yu, Danielle L. Mowery	2020	Journal	[79]
S65	A Privacy-Preserving and Standard-Based Architecture for Secondary Use of Clinical Data	Mario Ciampi , Mario Sicuranza, Stefano Silvestri	2022	Journal	[80]
S66	SemEHR: A general-purpose semantic search system to surface semantic data from clinical notes for tailored care, trial recruitment, and clinical research	Honghan Wu, Giulia Toti, Katherine I Morley, Zina M Ibrahim, Amos Folarin, Richard Jackson, Ismail Kartoglu, Asha Agrawal, Clive Stringer, Darren Gale, Genevieve Gorrell, Angus Roberts, Matthew Broadbent, Robert Stewart, Richard Jb Dobson	2018	Journal	[81]
S67	Applicability of Machine Learning Methods to Multi-label Medical Text Classification	Iuliia Lenivtceva, Evgenia Slasten, Mariya Kashina, Georgy Kopanitsa	2020	Conference	[82]
S68	A mobile health monitoring-and-treatment system based on integration of the SSN sensor ontology and the HL7 FHIR standard	Shaker El-Sappagh, Farman Ali, Abdeltawab Hendawi, Jun-Hyeog Jang, Kyung-Sup Kwak	2019	Journal	[83]
S69	Modeling and validating HL7 FHIR profiles using semantic web Shape Expressions (ShEx)	Harold R. Solbrig et al.	2017	Journal	[84]
S70	Structurally Mapping Healthcare Data to HL7 FHIR through Ontology Alignment	Athanasios Kiourtis et al.	2019	Journal	[85]
